# Genome-Wide Association Analysis Identified ANXA1 Associated with Shoulder Impingement Syndrome in UK Biobank Samples

**DOI:** 10.1534/g3.120.401257

**Published:** 2020-07-20

**Authors:** Bolun Cheng, Yujie Ning, Chujun Liang, Ping Li, Li Liu, Shiqiang Cheng, Mei Ma, Lu Zhang, Xin Qi, Yan Wen, Feng Zhang

**Affiliations:** Key Laboratory of Trace Elements and Endemic Diseases, Collaborative Innovation Center of Endemic Disease and Health Promotion for Silk Road Region, School of Public Health, Health Science Center, Xi’an Jiaotong University, 710061, P. R. China

**Keywords:** Shoulder impingement syndrome (SIS), Genome-wide association study (GWAS), PLINK, Gene set enrichment analysis (GSEA)

## Abstract

Shoulder impingement syndrome (SIS) is a common shoulder disorder with unclear genetic mechanism. In this study, Genome-wide Association Study (GWAS) was conducted to identify the candidate loci associated with SIS by using the UK Biobank samples (including 3,626 SIS patients and 3,626 control subjects). Based on the GWAS results, gene set enrichment analysis was further performed to detect the candidate gene ontology and pathways associated with SIS. We identified multiple risk loci associated with SIS, such as rs750968 (*P* = 4.82 × 10^−8^), rs754832 (*P* = 4.83 × 10^−8^) and rs1873119 (*P* = 6.39 × 10^−8^) of *ANXA1* gene. Some candidate pathways have been identified related to SIS, including those linked to infection response and hypoxia, “ZHOU_INFLAMMATORY_RESPONSE_FIMA_DN” (*P* = 0.012) and “MANALO_HYPOXIA_UP” (*P* = 5.00 × 10^−5^). Our results provide novel clues for understanding the genetic mechanism of SIS.

Shoulder impingement syndrome (SIS) is a common shoulder disorder which has been considered as the most frequent cause of shoulder pain (Falla *et al.* 2003). The estimated prevalence of shoulder complaints is 7–34% in the general population. SIS is the most commonly reported, accounting for 44–65% of all shoulder pain complaints (De Yang Tien and Tan 2014). It is commonly characterized as recurrent and chronic, with various pathogenic factors including acromial morphology, muscle imbalance, capsular laxity or tightness, rotator cuff muscle weakness, degeneration and inflammation of the tendons or bursa, dysfunctional glenohumeral and scapulothoracic kinematics (Windt *et al.* 1996; Conroy and Hayes 1998). SIS has serious influences on the economy due to its high treatment costs and to losses incurred through absence from work (Brox *et al.* 1993).

Previously, the researches of SIS mainly focused on clinical aspects including surgical treatment and kinesitherapy (Henkus *et al.* 2009; Haahr and Andersen 2006). On the pathophysiological areas, SIS has multiple functions, degradation and mechanical causes (Garving *et al.* 2017). The SIS impingent hypothesis postulated a pathophysiological mechanism, different shoulder joint structures producing mechanical conflicts (Garving *et al.* 2017). A Genome-wide Association Study (GWAS) was recently performed to identify the genetic variants associated with neck or shoulder pain in the UK Biobank cohort, and three genetic loci were identified associated with neck or shoulder pain, such as rs2049604 in *FOXP2* gene and rs62053992 in *LINC01572* gene (Meng *et al.* 2020). However, the molecular mechanisms of SIS are rarely studied and the genetic pathogenesis of SIS is still unclear.

In recent years, increasingly attention is focused on GWAS, an effective tool for studying the relationship between complex diseases and genetic susceptibility (Manolio *et al.* 2009). This technique applies millions of single nucleotide polymorphisms (SNPs) from the genome to perform case-control association analysis as markers and screens the SNPs in the whole genome to determine the susceptible sites and regions associated with diseases (Nicolae *et al.* 2010). Traditional GWAS focuses on single SNPs/genes that have a strong statistical association with phenotypic traits. Considering the potential biological interactions of the tested genes, a number of tools for downstream gene set enrichment analysis of GWAS results are widely used, such as GSEA (Wang *et al.* 2007) and FUMA (Watanabe and Taskesen 2017).

Gene set enrichment analysis (GSEA), also known as functional enrichment analysis, has been developed to identify functionally related genes that are significantly enriched in concentrations of genes that are signaly associated with complex diseases or traits (Du *et al.* 2018). Since GSEA integrates the joint genetic effects of multiple genes and prior information about biological processes (such as pathways), it shows a powerful function of biological interactions for pathogenetic research, especially for complex diseases or traits determined by a set of genes with moderate or weak genetic effects (Wang *et al.* 2007; Zhang *et al.* 2014).

To the best of our knowledge, no effort has paid to explore the genetic mechanism of SIS. Therefore a GWAS of SIS in UK Biobank was performed in this study. The GWAS results were further performed with GSEA to detect the candidate gene ontology and pathways associated with SIS. Our results may provide a new clue for understanding the molecular genetic mechanisms in the development of SIS.

## Materials and Methods

### UK BioBank samples

For the GWAS, we used the UK Biobank resource, a prospective cohort study including approximately 500,000 candidates from the UK, aged between 40 and 69 years, who have had whole-genome genotyping undertaken and have allowed the linkage of these data with their patient records (Bycroft *et al.* 2018). Briefly, participants provided self-reported information and collected blood samples for biochemical tests, genotyping, and physical measurements, as described previously (Bycroft *et al.* 2018). Individuals were linked retrospectively and prospectively to the National Health Service’s Hospital Episode Statistics database. The 9^th^ and 10^th^ revisions of the International Classification of Diseases (ICD) were used to define cases based on inpatient Hospital Episode Statistics data. Specific for this study, 3,626 SIS cases were identified according to the ICD10 diagnoses (Data Field: 41202) from the UK Biobank. Among the remaining samples, 3,626 samples were randomly selected as normal controls after excluding osteoarticular disorders according to the ICD 10^th^. Finally, a total of 7,252 participants from the UK Biobank cohort were included in this study (Table 1).

**Table 1:** Summary characteristics of the study populations

		Case	Control
Gender	Male/Female	1977/1649	1916/1710
Age (years)	Mean (SD)	57.94 (7.40)	56.90 (7.96)

### Genotyping

Genotyping details for UK Biobank participants have been reported previously (Bycroft *et al.* 2018). Briefly, 49,950 candidates were genotyped using the UK BiLEVE Axiom Array (807,411 markers) and 438,427 candidates were genotyped using UK Biobank Axiom Array (825,927 markers); the two arrays share about 95% of their marker content. Genotypes were called from the array intensity data and 106 batches of approximately 4700 samples were using a custom genotype-calling pipeline.

### Genome-wide association study of shoulder impingement syndrome

The GWAS of SIS were performed using 7,254 participants of European ancestry in the UK Biobank. All the subjects are unrelated self-reported White. The unrelated subjects were generated with KING software (http://people.virginia.edu/∼wc9c/KING/) (An *et al.* 2003). The kinship coefficients estimated by KING, and the option “–min” to apply the same cut-off for 3^rd^ degree (2 × 1/2^(9/2)^ = 0.088). The subjects who had a self-reported sex inconsistent with the genetic gender, who were genotyped but not imputed or who withdrew their consents were removed. A logistic regression model (implemented in PLINK2) assuming additive genetic effects was used for association analysis, adjusting for age, sex, PC1, PC2 and PC3 as covariates (Purcell *et al.* 2007). For quality control, we removed the SNPs with a call rate < 90%, SNP-level missingness (the number of individuals lacking information on a specific SNP is missing in the sample) > 0.1, individual-level missingness (the number of SNPs that is missing for a specific individual) > 0.1, minor allele frequency (MAF) < 0.01 and Hardy-Weinberg equilibrium (HWE) *P* value < 1.0 × 10^−3^. The Manhattan plot of GWAS results was drawn by R (https://www.r-project.org/). The quantile-quantile (QQ) plot was made by FUMA (Watanabe and Taskesen 2017).

### Linking GWAS findings to eQTL loci

GTEx eQTL dataset were used to identify genes with eQTL association for further investigation in the effect of gene variation on gene expression (GTEx Consortium *et al.* 2017). The identified GWAS SNPs were linked to eQTL loci from GTEx databases and then further evaluated for SIS related gene expression in our GWAS findings.

### Gene set enrichment analysis

The obtained GWAS summary data of SIS SNP were further subjected to gene set enrichment analysis (Wang *et al.* 2007; Subramanian *et al.* 2005). The detailed analytical procedures can be found in a published study (Liu *et al.* 2010). Briefly, four public pathway databases were utilized to generate a collection of gene ontology terms and pathways for testing in this study, which include BioCarta pathway database (http://www.biocarta.com/genes), Kyoto Encyclopedia of Genes and Genomes (KEGG) pathway database (http://www.genome.ad.jp/kegg/pathway.html), Ambion GeneAssist Pathway Atlas (http://www.ambion.com/tools/pathway), and Gene Ontology (GO) database (http://www.geneontology.org). A total of 2,850 gene ontology terms and pathways were analyzed in this study. 5,000 permutations were conducted to obtain the empirical distribution for the statistical tests. The enrichment scores and the empirical distribution were normalized to account for the different sizes of gene sets for each analyzed pathway databases. For each pathway gene sets, the empirical *P* value was calculated based on the observed normalized enrichment scores and the empirical distribution (Wang *et al.* 2007). Significant pathways were identified at empirical *P* values < 0.05.

### Data availability

The GWAS data (Category 100314) used in this study are available in http://biobank.ctsu.ox.ac.uk/crystal/label.cgi?id=100314. The UK Biobank resource can be found in http://www.ukbiobank.ac.uk/scientists-3/genetic-data/. Identification of SIS cases were followed to the ICD10 diagnoses (Data Field: 41202) from the UK Biobank in http://biobank.ctsu.ox.ac.uk/crystal/field.cgi?id=41202. Genome-wide significant loci associated with SIS are summarized in Table S1. Significant pathways associated with TWAS of SIS are summarized in Table S2. Quantile-quantile (QQ) plots of observed against expected *P* values for SIS is shown in Figure S1. All the supplementary materials including the GWAS summary statistics of SIS are available at figshare: https://doi.org/10.25387/g3.12477842.

## Results

### GWAS results of SIS

**Figure 1:**
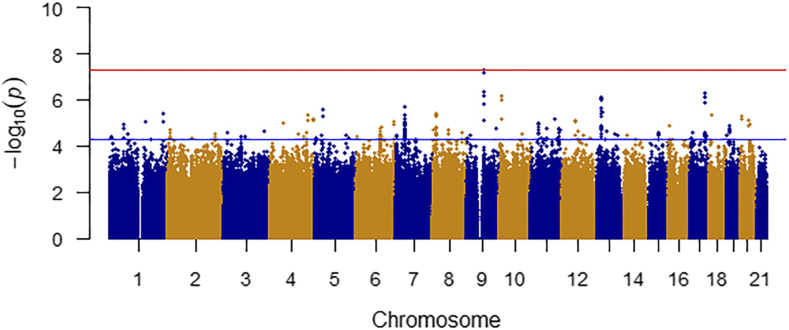
Manhattan plot for shoulder impingement syndrome in UK. Biobank Genomic coordinates are displayed along the X-axis, with the negative logarithm of the association *P* value for each SNP displayed on the Y-axis, meaning that each dot on the Manhattan plot signifies a SNP. The red line indicates the P-value threshold for genome-wide significance (P < 5 × 10^−8^) while the blue line indicates P-value threshold for suggestive significance (P < 5 × 10^−5^).

**Figure 2:**
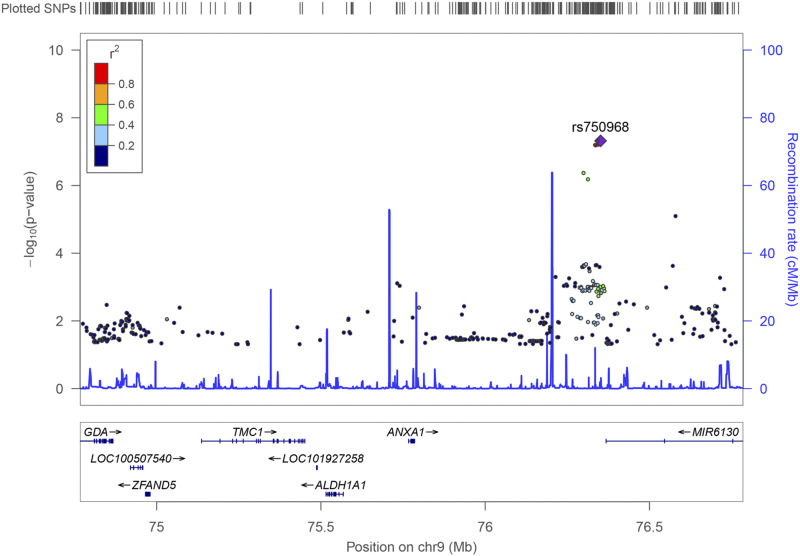
LocusZoom plots for shoulder impingement syndrome gene *ANXA1*. Association results for SNPs as a function of genomic distance (chromosomal position from National Center for Biotechnology Information build hg19) for *ANXA1*. The display range is chr9: 74766780−76785307. The top line shows genomic coverage at the locus, with each vertical tick representing the imputed SNPs. Purple diamond indicate SNP at the locus with the strongest association evidence. Each point represents a SNP. Bottom panel shows genes at each locus as annotated in the UCSC Genome Browser Annotation Database.

After controlling covariates including age, sex, PC1, PC2 and PC3, no statistical difference associations were observed in age, sex, PC1, PC2 and PC3 between SIS cases and controls (*P* > 0.05). Figure 1 presents the Manhattan plot of GWAS results of SIS. The plot is commonly used in GWAS to display significant SNPs (Gibson 2010). As shown, the strongest associations have the smallest *P* values; their negative logarithms will be the greatest. Significant associations were detected at the rs750968 (*P* = 4.82 × 10^−8^) and rs754832 (*P* = 4.83 × 10^−8^) of *ANXA1* gene on chromosome nine (Figure 2). Additionally, 4 SNPs of *ANXA1* showed suggestive association signals, including rs1873119 (*P* = 6.39 × 10^−8^), rs2130118 (*P* = 6.46 × 10^−8^), rs7029105 (*P* = 6.66 × 10^−8^), and rs7874184 (*P* = 4.31 × 10^−7^). We identified several candidate locus with suggestive association signals, such as the rs9987487 (*P* = 4.63 × 10^−5^) of *PLGRKT* gene on chromosome nine, and rs149598250 (*P* = 3.15 × 10^−5^) of *PIK3AP1* gene on chromosome ten (Table 2). The linked genes between identified SNPs of GWAS and eQTL loci of GTEx database are shown in Table S1. For example, *PLGRKT* (*P*_GWAS_ = 4.63 × 10^−5^, *P*_eQTL_ = 8.90 × 10^−7^), *TEKT3* (*P*_GWAS_ = 2.66 × 10^−5^, *P*_eQTL_ = 7.30 × 10^−7^) and *LIN28B* (*P*_GWAS_ = 2.94 × 10^−5^, *P*_eQTL_ = 2.40 × 10^−12^).

**Table 2:** List of candidate loci identified by GWAS of shoulder impingement syndrome

SNP	CHR	Gene	EA	OR	SE	P
rs750968	9	ANXA1	G	0.833	0.033	4.82× 10^−8^
rs754832	9	ANXA1	A	0.833	0.033	4.83× 10^−8^
rs1873119	9	ANXA1	A	0.835	0.033	6.39× 10^−8^
rs2130118	9	ANXA1	T	0.835	0.033	6.46× 10^−8^
rs7029105	9	ANXA1	T	0.835	0.033	6.66× 10^−8^
rs9987487	9	PLGRKT	A	1.145	0.033	4.63 × 10^−5^
rs149598250	10	PIK3AP1	C	2.185	0.188	3.15 × 10^−5^

Note: CHR = chromosome; EA = effect allele; OR = odds ratios; SE = standard error; P = *P*-value.

### Gene set enrichment analysis

Among all of the 2,850 pathways analyzed by this study, the “MANALO_HYPOXIA_UP” was the most significant associated signal for SIS in our analysis (*P* = 5.00 × 10^−5^, FDR = 0.018). We also found several candidate pathways for SIS, such as “BECKER_TAMOXIFEN_RESISTANCE_DN” (*P* = 1.00× 10^−4^, FDR = 0.018), “KEGG_NEUROACTIVE_LIGAND_RECEPTOR_INTERACTION” (*P* = 1.00× 10^−5^, FDR = 0.015) and “IZADPANAH_STEM_CELL_ADIPOSE_VS_BONE_DN” (*P* = 1.49× 10^−5^, FDR = 0.018). Table 3 summarized the ten top-ranking pathways detected by this study. Detailed results of our analysis can be found in Table S2.

**Table 3:** Top ten of significant pathway associated with shoulder impingement syndrome

Pathway	*P* value	FDR q value
MANALO_HYPOXIA_UP	5.00× 10^−5^	0.018
NIKOLSKY_BREAST_CANCER_8Q12_Q22_AMPLICON	5.00× 10^−5^	0.021
BECKER_TAMOXIFEN_RESISTANCE_DN	1.00× 10^−4^	0.018
DELYS_THYROID_CANCER_DN	1.00× 10^−5^	0.002
DING_LUNG_CANCER_BY_MUTATION_RATE	1.00× 10^−5^	0.018
GARGALOVIC_RESPONSE_TO_OXIDIZED_PHOSPHOLIPIDS_GREY_UP	1.00× 10^−5^	0.035
KEGG_CALCIUM_SIGNALING_PATHWAY	1.00× 10^−5^	0.018
KEGG_NEUROACTIVE_LIGAND_RECEPTOR_INTERACTION	1.00× 10^−5^	0.015
WEI_MIR34A_TARGETS	1.00× 10^−5^	0.018
IZADPANAH_STEM_CELL_ADIPOSE_VS_BONE_DN	1.49× 10^−5^	0.018

## Discussion

In this study, we conducted a GWAS of SIS using a large data set from UK biobank samples. GWAS results were further performed with GSEA to detect candidate gene ontology and SIS related pathways. We detected several candidate genes and gene sets for SIS, such as *ANXA1* and *PLGRKT*.

One vital finding of this study is the two significant risk SNPs rs754832 and rs750968, both of them were near to the *ANXA1* (Annexin A1) gene on chromosome nine. This gene encodes a membrane-localized protein that inhibits phospholipase A2 and has anti-inflammatory activity (Cardin *et al.* 2017). A research identified the regulation of the inflammation by regulating the clearance of neutrophils by macrophages in mouse bone marrow as a critical regulator role for the anti-inflammatory molecule *ANXA1* (Dali *et al.* 2012). Moreover, *ANXA1* is essential to regulate bone marrow mesenchymal stem cells (BMSCs) proliferation and osteogenic differentiation (Pan *et al.* 2015). Likewise, Liang *et al.* investigated the effects of silencing *ANXA1* gene on proliferation and osteogenic differentiation of rabbit BMSCs, and found that silencing *ANXA1* gene could reduce those capacities of BMSCs, which may be one of mechanisms underlying osteoarticular diseases (Liang *et al.* 2012). Our results suggest that *MIR6130* is another gene near the top SNP rs750968. *MIR6130* (microRNA 6130), alias as hsa-mir-6130, is one of the microRNAs that coordinately regulated in rheumatic heart disease patients with mitral valve stenosis (Lu *et al.* 2018). However, the relationship between *MIR6130* and SIS remains unclear.

In this study, another identified candidate genes for SIS was *PLGRKT* (Plasminogen Receptor with A C-Terminal Lysine) which involved in regulation of inflammatory response; regulated monocyte chemotactic migration and matrix metalloproteinase activation, such as of *MMP2* and *MMP9* (Lighvani *et al.* 2011). It has been demonstrated that Plasminogen (PLG) and the plasminogen receptor, *PLGRKT*, play an important role in macrophage recruitment in the inflammatory response, and regulated inflammation via the interplay between PLG and its receptors (Miles *et al.* 2014). Miles *et al.* evaluated the effect of *PLGRKT* deletion on inflammation, and demonstrated that *PLGRKT* was required for plasminogen binding and macrophage migration *in vivo* (Miles *et al.* 2017). Likewise, *PLGRKT* involved in regulation of inflammatory response; regulates matrix metallproteinase activation, which may damage cells by digesting the ECM (Erlykina *et al.* 2015).

GSEA detected several candidate biological gene sets for SIS, such as “ZHOU_INFLAMMATORY_RESPONSE_FIMA_DN” (genes down-regulated in macrophages by *P.gingivalis FimA* pathogen), which was related to inflammatory response. This gene set up-regulated in macrophages by lipopolysaccharide (LPS) and can lead to an inflammatory condition (Zhou and Amar 2007). Several investigators have suggested that fimbriae of *P.gingivalis* can trigger the production of proinflammatory mediators in human macrophages (Nakagawa *et al.* 2002). Besides, LPS in *P.gingivalis* could promote the antibacterial responses of macrophages, and simultaneously cause detrimental lesion in infected tissues due to enhanced local inflammatory reactions (Mancuso *et al.* 2007). Therefore, this gene set may affect the development of SIS by regulating inflammatory responses, but needs further verification.

“MANALO_HYPOXIA_UP” (genes up-regulated in response to both hypoxia and overexpression of an active form of HIF1A), another candidate biological gene sets associated with SIS, played a vital role in the pathogenesis of osteoarthritis (OA) and rheumatoid arthritis (RA) (Johnson *et al.* 2000; Brouwer *et al.* 2009; Pfander *et al.* 2006). However, the molecular mechanism of hypoxic-induced articular lesions in osteoarthrosis remains controversial. A variety of hypotheses on the role of hypoxia in the pathogenesis of osteoarthrosis, such as abnormal energy metabolism and immune inflammation have been proposed in previous studies (Pfander and Gelse 2007). For example, the molecular mechanism of hypoxia-induced joint injury may be partly due to the increase in production of ROS under hypoxia, which can accelerate protein oxidation, and induce mitochondrial injury and apoptosis (Zhang *et al.* 2011). Based on these evidences above, we suggested that hypoxia might play an important role in the pathogenesis of SIS.

The gene set enrichment researches are receiving increasing attention in genetic study of human complex diseases. Although this is the first molecular mechanism study in SIS combining GWAS and gene set enrichment analysis, there are still several limitations need to be addressed. First, the sample size of SIS GWAS is not large enough. Additionally, our analysis results need to be verified in experimentation via collecting sufficient SIS samples. Consequently, our study results should be interpreted with caution and further studies are needed to confirm the findings and reveal the potential roles of identified locus and pathways in the development of SIS.

In conclusion, we conducted GWAS for SIS, and then performed a gene set enrichment analysis based on GWAS results. Finally, a group of significant difference genes and gene sets within SIS were identified, which may provide novel clues for revealing the complex genetic mechanism of SIS.
